# Eating Behaviors in Postpartum: A Qualitative Study of Women with Obesity

**DOI:** 10.3390/nu10070885

**Published:** 2018-07-10

**Authors:** Débora Bicudo Faria-Schützer, Fernanda Garanhani Surita, Larissa Rodrigues, Egberto Ribeiro Turato

**Affiliations:** 1 Postgraduate Program in Obstetrics and Gynecology, Department of Obstetrics and Gynecology, School of Medical Sciences, University of Campinas, BR-13083881 Campinas, São Paulo, Brazil; surita@unicamp.br (F.G.S.); rodrigues-larissa@uol.com.br (L.R.); erturato@uol.com.br (E.R.T.); 2 Department of Medical Psychology and Psychiatry, School of Medical Sciences, University of Campinas, BR-13083881 Campinas, São Paulo, Brazil

**Keywords:** pregnancy, nutrition, obesity, eating behaviors, qualitative study, low- and middle-income countries

## Abstract

In postpartum, women experience major changes in their lives; they are forced to deal with new internal and external demands for attention and care for themselves and the baby. Postpartum feeding also suffers changes in this stage of life, because women find more barriers to healthy eating, which can put them at greater risk of overweight or obesity. This is a qualitative study, through in-depth semi-directed interviews in an intentional sample with postpartum women with obesity, closed by saturation and qualitative content analysis. Sixteen women were included. Three categories emerged from this analysis: (1) from pregnancy to postpartum: changes in body and eating behavior; (2) eating to fill the void of helplessness felt during the postpartum period; and (3) breastfeeding and baby feeding. Women with obesity eat to relieve unpleasant feelings during the postnatal period. The postpartum period is an opportune moment to introduce long-term changes in the eating behaviors and mental wellbeing of these women. Healthcare teams need to restructure to provide more focused follow-up care for women with obesity during the postnatal period in terms of their physical and emotional health.

## 1. Introduction

In postpartum, new adjustments are required for the woman to adapt to the baby’s care routine and gradually return to the pre-gravid physiological and metabolic conditions. Due to this, women become more susceptible to physical and psychological complications [1,2,3,4]. It is impossible to dissociate postpartum from the experience of motherhood. The process of construction of motherhood begins in stages that precede gestation. However, it is from childbirth that women will realistically experience “being a mother”. From a psychological point of view, postpartum can be characterized by ambivalent feelings, such as euphoria and relief, fear of not being able to nurse, fear for the baby’s health; fear of not being able to take care and respond to the baby’s needs; or not being able to practice motherhood properly [5,6]. Motherhood brings out important issues to the psychological and social identities of women. They experience major changes in their lives that requires physical and psychosocial adjustments, and that will be reflected in the way each woman constitutes herself as a whole [7].

The transformations that begin in puerperium, with the purpose of restoring a woman’s body to a non-pregnant situation and adapting it to motherhood also include alterations in her brain structure, which can grant adaptive advantages to motherhood, such as making it easier for a mother to recognize her child’s needs [8].

Postpartum feeding also suffers from this new condition [9,10] and women find more barriers to healthy eating, which can put them at greater risk of overweight or obesity [11]. Vieira et al. [9] demonstrate that most puerperal women presented with unbalanced nutrition, influenced by cultural, psychological, and economical factors.

Women who initiate pregnancy with obesity present higher food insecurity, poorer quality of food and weight gain in the following year after childbirth [12,13,14,15]. The etiology of obesity is complex and multifactorial [16,17,18,19]. Obesity is strongly influenced by biology, however, understanding its association with psychological factors is paramount for clinical management [19]. From a psychodynamic perspective, obesity can be seen as an expression of a certain psychic structure, encompassing emotional struggles and difficulties in interacting with the environment that are experienced in early stages of human development [20].

Gender issues also influence nutrition directly. Most Western societies assign the responsibility of selecting, preparing, and sharing the food among the family to women, in addition to childcare. Even though they are responsible for preparing the food, women usually do that by taking into account only their husbands’ and children’s tastes, and not their own [21]. Post-gestational weight retention in mothers with obesity is also associated with psychological discomfort during pregnancy [22]. Women who become pregnant when overweight or obese have a greater association between food anxiety and weight gain after childbirth. These women need a multidisciplinary approach, including psychological and nutritional aspects [12].

Evaluation of psychological aspects of women with obesity during the postpartum period is recommended, in order to improve the mother-baby relationship, promote breastfeeding, and promote the integral health of women in the medium- and long-term [9,16,18]. Nourishment is related not only to nutrition, but also to the exchange, to the contact with the other, and to the entire relationship of love and care that is established between mother and child. Nourishment and the feeling of pleasure/displeasure leave indelible marks on the individual’s psyche from the first breastfeeding [23].

The preceding stage, during and after pregnancy, should be seen as a “window of opportunity” to minimize short-and long-term health risks for women and their children [24,25]. Weight-watching and caring about nutrition are issues of interest to women in postpartum [26,27].

The present study emerged from the demands of previous studies carried out in this same Health Service and the professionals who provide prenatal care and perform postpartum review consultations. This study aimed to explore the psychological meanings that women with obesity attribute to their diet during the postnatal period: what they eat, whether they have a self-care regimen, their relationships with themselves and their baby, as well as their motivation to adopt a new lifestyle.

## 2. Materials and Methods

### 2.1. Study Design

A clinical-qualitative method with content analysis was adopted, which was composed of three characteristics: (1) existentialist attitude: value attributed to the angst and anxieties by the diseased; (2) clinical attitude: acceptance of suffering; (3) psychodynamic attitude: consideration that subconscious aspects are present in the interviewer-interviewee relationship and in the subject discussed [28,29,30].

### 2.2. Data Collection and Sample

A four-month period of observation of medical and nursing consultations, subgroups, and clinical discussions was performed at the data collection site, the Woman’s Hospital Post-partum Outpatient Clinic of the University of Campinas, a university hospital, located in Southeastern Brazil. That period is referred to in the qualitative research as acculturation period. All information from that stage was recorded in the field diary and used to formulate interview questions.

The participants were selected and invited to participate in the study from the data recorded in the medical records. Participants were approached between April and August 2017 on the same day as their consultation, with each woman being approached intentionally based on the following criteria: older than 18 years of age; up to six months postpartum; and BMI ≥ 30 kg/m^2^ before pregnancy. Women who opted not to breastfeed were excluded. The sample was closed by the information saturation criterion [31], after discussion and validation with two research groups from the Faculty of Medical Sciences: Clinical-Qualitative Research Laboratory (LPCQ) and Reproductive Health and Healthy Habits (SARHAS).

Based on the expectation that the interviewees would speak as openly as possible about their experiences in relation to aspects of feeding, weight, and feelings during postpartum, semi-structured individual interviews with the following open question were used to collect the data: Tell me about how you have been feeling since your baby was born?

The interview that began this way was planned based on a script with questions determined by the aim of the study, the acculturation period and the review of scientific literature, and that provided support for the researcher: Do you feel you have been taking care of yourself since then? How has your nutrition been since then? Do you think about or take care of your own nutrition? Have you thought about or done anything regarding your weight during pregnancy? Has any of that changed now? How do you see your body and its transformations since the baby was born? How do you feel you have been taken care of? Is there anyone helping you out at home? How do you feel while breastfeeding? When you are eating, do you think about breastfeeding? What challenges do you face at the moment in order to keep a healthy diet? What does food mean to you?

These were more cues than questions that were used in the appropriate moment of the interview, in case the interviewees had not broached these topics spontaneously.

All interviews were performed (by the first author DBFS) in a private room, recorded and transcribed, with an average duration of 440 min, adding the time of all interviews. Only one single interview was conducted with each participant, lasting an average of 27 min each. Interviewees spoke freely about their experiences and feelings of obesity and the postpartum period. Observational data and self-observation of the interviewer were recorded in a field diary, during and after each interview.

### 2.3. Data Analysis

Content analysis [28,32] was used. This form of analysis involves a process of organization, comprising seven steps, applied by the authors (according to their initials): (1) editing material—“transcribed corpus” (LR); (2) floating reading—the researcher become impregnated by the reported life experience (DBFS and LR, separately); (3) construction of the units of analysis—the researchers capture meanings, select the fragments, and develop initial reflections. The creative part of the researcher’s work is focused on the development of meanings (DBFS and LR); (4) construction of codes of meaning—creation and structuring of the first codes of meaning (all authors); (5) construction of categories—organization of the material for analysis of all participants with a view to grouping (DBFS, LR), with consensus obtained after discussion with FGS; (6) discussion—a dialogue with the available literature (DBFS, FGS, LR); and (7) validity—validity involves the critical reflection on the processes carried out in each step (all authors and the COREQ-Checklist).

The Ethics Committee of the University of Campinas and Brazilian National Board of Health approved this research, under number CAAE: 62565116.3.0000.5404 and took into account the requirements laid down by the Brazilian National Board of Health. The validity period of the approval is four years and the researcher must keep the research data on file, under his responsibility, for a period of five years after the end of the research. We have received written informed consent from all participants.

## 3. Results

Sixteen interviews were conducted. Some characteristics of the interviewees are recorded in Table 1.

### 3.1. From Pregnancy to the Postpartum Period: Changes in Body and Eating Behavior

As in pregnancy, the postpartum period places a woman into contact with food and their body, yet in a different way. Their relationship with life, activities of daily living, and with time itself changes, with their feelings become more intense and ambiguous. While pregnancy is full of expectations about motherhood and life with a baby, the postpartum period cements action and care for the newborn.

Even in pregnancy I said, I can continue as normal, I had a shock when the baby was born, and I saw that now another person depended totally on me.

*(Interviewee 6)*

Those interviewees knew of their diagnosis of obesity and were aware of it being bad for their health, yet they did not know how to combat it. There was great discomfort and sadness regarding their weight and body.

When I weigh myself it is one of the saddest moment for me.

*(Interviewee 1)*

I’m not feeling very good about my body. I cannot cope well with it; it makes me feel really bad. My biggest worry is that I’ll get fat all over again.

*(Interviewee 15)*

They spoke about the awareness that, in order to change, the initiative should come from them. They compared obesity to addiction or a personality disorder. They feel the “weight” of the body emotionally, but as if that is part of who they are, or something far than themselves can face.

I say that is it shameful, you know that person that smokes, that could stop smoking but doesn’t want to, it’s shameful…. I could stop eating, but I don’t want to, I have to have this pleasure in life ...

*(Interviewee 6)*

Eating habits and weight are not principal issues at this point in life. These women cannot focus their concentration on this during the postpartum period and seem to summon the healthcare team to ignore it too. To lose weight or to include healthy habits in your life must be desired by the individual involved.

Yes, I’m sorry ... it’s that ... I want to lose weight.... let’s see if I can go out walking now that I’m at home ... But now he’s tiny, right? Forget it....

*(Interviewee 2)*

I don’t have time, I do not even think about it.

*(Interviewee 5)*

Pregnancy provided an opportunity to consider eating behaviors, resulting in changes during that period. After childbirth, however, they returned to their old eating habits.

I’m trying to take care of myself so I do not get fat ... I weighed 100.1 kilos ... it made me very sad, as I had said that I did not want to reach three digits again ... I realized during my pregnancy that eating is my problem, so if I could do it pregnant, why not now?

*(Interviewee 3)*

As for weight gain during the postpartum period, interviewees with more than one child reported that retention of weight gained during pregnancy led to obesity.

Ah, with my firstborn I was much thinner, but then I gained weight and could not lose it anymore ...

*(Interviewee 8)*

Additionally, the vast majority of interviewees reported that shortly after birth they lost weight, yet after the first six months, they had in fact gained weight.

After he was born I lost weight, however, I stayed indoors for months, so I got fatter, (...) There was no routine, and at home I eat, I get nervous, I eat all the time, I never ran out of candy, so I got fat.

*(Interviewee 8)*

The discourse of the interviewees, besides weight gain, shows us how the psychological factors contribute to an inadequate diet or even a binge eating and, consequently, weight gain.

### 3.2. Eating to Fill the Helplessness Experienced during the Postpartum Period

This category refers to the often contradictory and intense feelings involved in the process of “becoming a mother”, and the resources these women with obesity have to deal with these feelings, which, in their case, involves food. This situation brings us to think of a food psychology, which is related to the influences that the feelings and the individual’s way of dealing with them brings to the habits and eating behaviors.

... There is nothing specific, you feel lost, you do not know why you’re crying.... you do not know why, it’s strange, I think it comes from the past and mixes with things of the present. I don’t know, I just know that I’m like this.

*(Interviewee 3)*

In all of the interviews, besides the typical tiredness experienced by the woman in the postpartum period, the burden of loneliness and helplessness was present. There is a sense of loss of individuality and they feel that their loved ones do not recognize this pain.

... When he cries too much ... you already did everything you could and do not know why he is crying... It’s mentally exhausting... I act as if nothing is happening so as not to get worked up anymore... And at night he wakes up to feed, two or three times before dawn ... and then 5:30 a.m. the alarm goes off.

*(Interviewee 3)*

Postpartum requires hard and intense psychological work. It is emotional work that involves feelings of letting a new person occupy the most important place in your life, including more than yourself. Women also have to deal with changes in psychological and social roles.

The baby takes center stage at home and in their emotions, which prevents these women from focusing on self-care.

Ah, so, it’s complicated, I ended up gaining weight sideways anyway, there’s hair, nails to do, so I don’t go out anymore, right? I just stay at home with him (the baby), so because of that it doesn’t make much of a difference, but we do miss that as women, you know?

*(Interviewee 4)*

During the postpartum period, there is a psychological responsibility of forsaking the place of being a daughter to effectively assume the role of mother (change of roles). These transformations are positive, yet they require a significant internal dialogue.

I start thinking, now I’m the mother, it’s me that has to face things, before it was my mother that tackled everything face on ... And now, I have to take initiative for everything ... I think it’s been good for me to grow up.

*(Interviewee 1)*

Postpartum psychological processes are felt and perceived by women with obesity, but they find it difficult to labor with these unpleasant feelings. Mental elaborations often bring unpleasant feelings, and the woman with obesity finds it difficult to labor with these frustrations and eats to feel more relieved and to compensate for these displeasures by the pleasure of food. The feeling of helplessness and loneliness remain and they develop eating to fill the void of postpartum.

The emotional responsibility, linked to the subjectivity of these women with obesity in the postpartum period, generates a great deal of anguish and discomfort. Food, in this case, becomes a source of pleasure, used to deal with unpleasant feelings during the postpartum period.

I arrive and want to eat, and I start searching for whatever I can find and it satisfies me. It provides profound happiness!

*(Interviewee 3)*

### 3.3. Breastfeeding and Baby Feeding

The interviewees expressed themselves in a divided manner about breastfeeding: loving or hating it. In this study many feelings were projected on to the baby, as if it were the baby expressing the opinions, not the mother:

My baby did not want to, he was disgusted which caused nausea. He snorted! He was anxious, did not want to feed, and cried for half an hour before feeding.

*(Interviewee 6)*

I thought it was wonderful, I said I wanted it, but then it was too late (to breastfeed).

*(Interviewee 13)*

Likewise, some of the gratification that the woman feels is associated to her experiences of pleasure and satisfaction with regards to eating behaviors:

And I like to breastfeed, it’s the thing that I like the most ... (laughs) ... Because it’s really wonderful, after they feed, they give that happy little smile, and you know that they are full.

*(Interviewee 1)*

The relationship between these women and food influences the baby’s eating behaviors. Psychological aspects, such as self-confidence to make adequate choices regarding ones’ own food, as well as that of their child, are low and often require the help of others.

So you have to set an example, I want to teach him the best way, my husband says: you will not be able to give them the best in terms of food, that will have to come from me.

*(Interviewee 6)*

The interviewees understood that breastfeeding is related to weight loss, however, they reported that this often left them more stressed and nervous. Rather than being an incentive, it became a complication or even a hindrance.

My mum kept saying: you don’t want to breastfeed, but breastfeeding is what will help you to lose weight. It just kept putting the pressure on me and I got irritated.

*(Interviewee 13)*

## 4. Discussion

Our results are in accordance with the literature in that during postpartum a new phase begins for women in their experiences and relations, very different from those perceived during pregnancy [33]. The careful eating and health habits suggested and supported by the prenatal care team that women with obesity have taken on during pregnancy were abandoned in postpartum. We understand that such abandonment is linked to internal and external influences experienced by women with obesity, which we highlight: (1) They feel uncomfortable with an obese body, but they do not know how to deal with their obesity; (2) Nutrition is not a central issue for a woman with obesity at that point in her life; (3) There is a return to old eating behaviors that have been developed since childhood/adolescence that are linked to obesity; (4) During postpartum there is a very present feeling of loneliness and helplessness, which strongly influences the eating behavior of women with obesity during that period.

It is perceived that some of those are intrinsic to that postpartum moment in a woman’s life. However, it is possible to discuss some aspects of these women’s health from psychological issues involved in the structure of obesity and its relation to nutrition.

Obesity, in itself, is a complex phenomenon that, as a health issue, demands deep, critical considerations. The study of individuals with obesity assumes that they have characteristics and psychological structures specific to people with obesity (intrapsychic elements) [34] and that obese people are usually subject to serious prejudice and discrimination in a number of everyday situations, including from health professionals (psychosocial elements) [7,34,35]. These psychological and psychosocial elements are important barriers to assess when it comes to healthy eating, and should be considered throughout the process of monitoring a woman with obesity during the postpartum period.

When women with pre-pregnancy obesity enter the postpartum period, they begin to feel responsible for their body habitus. Additionally, the influence of psychological and motivational issues regarding their eating behaviors is considered indisputable, as they have no control, which provokes a feeling of weakness and disbelief in their own abilities to transform their eating behaviors in the long-term. Furthermore, these women report disappointment in a derogatory or self-harming manner. Data from the present study on coping with helplessness are also consistent with another study [36], which demonstrated that BMI before pregnancy and postpartum are related and positively correlated with the overall severity index of body discomfort.

Interviewees talked about pregnancy as a possible moment to reconsider their eating behaviors, resulting in some behavioral changes, which could not be maintained after childbirth, with a return to old behaviors early on leading to weight gain. These results suggest the importance of specific attention to these issues during the postpartum period. These findings corroborate previous studies, which the attention and interventions of the health team should continue into postpartum so that the adoption of new habits is guaranteed [37].

Women suffer the highest cost as a direct result of reproduction, including pregnancy, lactation, and childcare. This has a major impact on women’s health, particularly those with obesity, due to the association between parity, obesity, and non-communicable diseases. The results of this study demonstrate that after the birth and, with a new daily routine already installed, the interviewees described that they began gaining weight once again and how difficult it was for them to keep healthier eating habits that had been adopted during pregnancy. These findings corroborate previous studies [12,38] that indicate that from three months postpartum some women have a greater weight due to food insecurities and food behaviors. This study suggests to postpartum being a favorable moment to address changes in eating behaviors, since some of these changes have already been experienced during pregnancy [30,39,40,41,42].

This study demonstrates how living with the feeling of helplessness, alongside the psychological changes that these women go through during their new tiring routine of baby care, causes changes in their eating behaviors. Previous studies have shown an unbalanced nutrition during the postpartum period, which is influenced by cultural, psychological, and economic factors [9,12]. The current study developed these issues, revealing that unpleasant postpartum feelings can cause a woman with obesity to acquire a psychological-eating pattern, in which food becomes a compensatory mechanism for her disheartened feelings, and where food acts as a drug-like escape valve [43,44].

The constant presence of mothers and mothers-in-law support a more balanced postpartum diet, as they often help with the preparation of healthier foods [42]. The results of the present study also showed the importance of familial involvement with the postpartum routine, helping to promote feelings of improved security and self-esteem, thus reducing the sense of discontentment. However, some interviewees pointed out that some familial relations might be perceived by them as intrusive and/or as if the family did not feel that those women were capable of taking care of themselves and of taking care of the baby by themselves. Interviewees reported that this could be a significant stressor, and that it induced negative emotions. It is important for us, as health professionals, to think about the limits of those interventions in order to help these women develop their autonomy. As health professionals, we should always encourage them and help them find the best way to learn what to do by themselves or how to ask for help when needed, always respecting the uniqueness of each woman and their contexts.

Socio-economic and cultural aspects of the relationship with the partner, as well as the cultural role of the woman-mother in Brazil saturated the reports obtained from the interviewees in this study. However, the study method chosen did not allow for more in-depth sociological studies.

Considering the woman from her point of view, as the protagonist of the experience, could be of use to the healthcare team in terms of promoting adherence to treatments and changes in eating habits, as well as to provide an opportunity to practice preventative measures with regards to weight and mental health.

The postpartum woman’s need to be looked after and supported is also reflected with regard to the healthcare team. The postpartum period is a moment that demands significant psychological attention with regard to the loss of her pre-maternity expectations. During that phase, oftentimes attentions are turned to the new member of the family and there is an expectation that the woman takes on the idealized maternal role quickly. That sentiment is confirmed by health promotion actions, which in this period are, in the great majority, centered around babycare and breastfeeding [7]. This study was able to identify how women with obesity deal with emotional peaks during the postpartum period, specifically that they eat to appease unpleasant feelings. The feelings experienced in the postpartum period are a “trigger” for binge eating. The relation of these women to the food is of compulsiveness and discharge of the anxiety experienced in the postpartum period. Our results are in accordance with the literature that postpartum begins a new phase for the woman in her experiences and that deserves the attention of the health professionals [37,45]. Those professionals that are willing to assist women with obesity during the postpartum period should be aware of the mothers’ emotional fragility, foster them, and give them support so that they overcome difficulties that arise from motherhood [46]. Just like the care that was perceived during the prenatal period, these women need to perceive that the postpartum care team is able to attend to their needs and motivate them to take care of themselves, especially in regard to eating and weight. Moreover, when needed, the team can take of that for them and encourage them to ask family members and friends for support, when they feel overwhelmed.

The results of this study also call attention to the type of care provided by the mother for her baby, since unhealthy food choices are often made, including when considering the weaning, which corroborates other studies that focus on the influence of food during the mother-child relationship [47,48].

This study sought to instrumentalize health professionals who accompany women in the postpartum period and/or call the attention of health professionals to the need for postpartum follow-up in women with obesity. Knowing what they feel and think can change their approach and clinical management. Our study indicates the need to include in the obesity treatments the psychological aspects, within a holistic view, that considers feeding and obesity as biopsychosocial phenomena.

## 5. Conclusions

The food and health care that women with obesity assumed during pregnancy and who were indicated and supported by the prenatal follow-up team were abandoned in the postpartum period. We understand that this abandonment is related to the internal and external motivations experienced by women with obesity, which we highlight the following: they feel uncomfortable with the obese body, but do not know how to deal with their obesity; food is not a central issue for women at this time of life. There is a return to old eating behaviors developed since childhood/adolescence; in postpartum there is a present feeling of solitude and helplessness, which influences the eating behavior; and caring for the baby becomes a priority than the care itself.

This study sought to instrumentalize the health professionals who accompany the woman in the postpartum period or to call the attention of the health professionals to the need for postpartum follow-up in women with obesity. Knowing what they feel and think can change the approach and clinical management. Health teams can be structured to improve care for this population, providing spaces for listening, welcoming, and counseling to these women and their environment.

## Figures and Tables

**Table 1:** Participants’ characteristics.

Participants	Age (Years)	Months Postpartum	Pre-Pregnancy Weight (Kg)	GWG (Kg)	Current Weight (Kg)	Current BMI (Kg/m^2^)
E1	22	5	93	4	93	36.3
E3	34	2	110	12	118	45.5
E3	33	5	118	-18	99	33.5
E4	23	2	102	6	94	31.8
E5	26	1	88	25	97	32.8
E6	23	4	120	-8	105	37.6
E7	34	2	90	6	91	34.7
E8	29	3	88	14	94	32.9
E9	27	1	95	5	79	31
E10	29	4	95	22	96	31
E11	43	2	110	8	99	34.3
E12	39	3	84	12	84	35.4
E13	23	5	140	−10	142	52.2
E14	29	2	85	4	79	31.2
E15	36	3	86	18	85	32.8
E16	20	2	82	10	82	30.5

GWG: Gestational Weight Gain, BMI: Body Mass Index. Three categories emerged from analysis: (1) from pregnancy to postpartum: changes in body and eating behavior; (2) rating to fill the void of helplessness felt during the postpartum period; and (3) breastfeeding and baby feeding.
